# 1,3-Bis(hydroxymethyl)harnstoff: MAK-Begründung, Nachtrag

**DOI:** 10.34865/mb14095kskd10_1ad

**Published:** 2025-03-31

**Authors:** Andrea Hartwig, MAK Commission

**Affiliations:** 1 Institut für Angewandte Biowissenschaften. Abteilung Lebensmittelchemie und Toxikologie. Karlsruher Institut für Technologie (KIT) Adenauerring 20a, Geb. 50.41 76131 Karlsruhe Deutschland; 2 Ständige Senatskommission zur Prüfung gesundheitsschädlicher Arbeitsstoffe. Deutsche Forschungsgemeinschaft, Kennedyallee 40, 53175 Bonn, Deutschland. Weitere Informationen: Ständige Senatskommission zur Prüfung gesundheitsschädlicher Arbeitsstoffe | DFG

**Keywords:** 1,3-Bis(hydroxymethyl)harnstoff, Nase, oberer Atemtrakt, Reizwirkung, Kanzerogenität, Formaldehydabspalter, Strukturanalogie, Keimzellmutagenität

## Abstract

The German Senate Commission for the Investigation of Health Hazards of Chemical Compounds in the Work Area (MAK Commission) has re-evaluated 1,3-bis(hydroxymethyl)urea [140-95-4] with regard to its carcinogenicity and germ cell mutagenicity classification, its ability to be absorbed through the skin, its sensitization potential and whether an occupational exposure limit value (maximum concentration at the workplace, MAK value) can be derived. Relevant studies were identified from a literature search. 1,3-Bis(hydroxymethyl)urea is a skin and eye irritant after prolonged exposure. The substance is a formaldehyde releaser and is expected to undergo rapid hydrolysis in aqueous solution. For this reason, the local irritation is attributed to the hydrolysis products formaldehyde and urea. Carcinogenicity, toxicity and genotoxicity of 1,3-bis(hydroxymethyl)urea in the upper respiratory tract or nose, the likely target organs, have not been investigated. The substance has mutagenic and clastogenic potential in vitro, presumably due to the release of formaldehyde. Formaldehyde was classified in Carcinogen Category 4 because it induces tumours in nasal tissues at concentrations that exceed their detoxification capacity. As a formaldehyde releaser, 1,3-bis(hydroxymethyl)urea could likewise be classified in Carcinogen Category 4. However, because it is not possible to derive a MAK value for 1,3-bis(hydroxymethyl)urea, the substance has been assigned to Carcinogen Category 2 with the footnote “Prerequisite for Category 4 in principle fulfilled, but insufficient data available for the establishment of a MAK or BAT value”. As there are no data for the systemic bioavailability of 1,3-bis(hydroxymethyl)urea and the formaldehyde that is released by hydrolysis in tissues, there is no experimental evidence that the formaldehyde reaches the germ cells. Therefore, 1,3-bis(hydroxymethyl)urea has been classified in Category 3 B for germ cell mutagens. There are no data investigating sensitizing effects in humans. A local lymph node assay in mice yielded negative results. Skin contact is not expected to contribute significantly to systemic toxicity.

**Table:** 

**MAK-Wert **	**–**
**Spitzenbegrenzung **	**–**
	
**Hautresorption**	**–**
**Sensibilisierende Wirkung**	**–**
**Krebserzeugende Wirkung (2024)**	**Kategorie 2^a)^**
**Fruchtschädigende Wirkung**	**–**
**Keimzellmutagene Wirkung (2024)**	**Kategorie 3 B**

**EKA**	**–**

Synonyma	Dimethylolharnstoff N,N'-Dihydroxymethylharnstoff
Chemische Bezeichnung (IUPAC-Name)	1,3-Bis(hydroxymethyl)harnstoff
CAS-Nr.	140-95-4
Formel	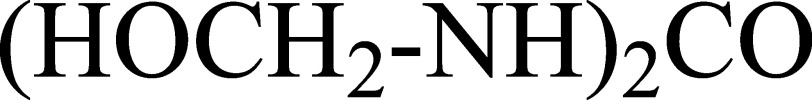
	C_3_H_8_N_2_O_3_
Molmasse	120,11 g/mol
log K_OW_	–3,15 (ber.; ECHA 2020)
Dampfdruck bei 20 bis 25 °C	< 1 × 10^–6^ hPa (OECD TG 104; ECHA 2020)
Hydrolysestabilität	k. A. (ECHA 2020)
Einsatzverbote	als Biozid (European Commission 2013; Hartwig und MAK Commission 2023 a)

^a)^ Voraussetzung für Kategorie 4 prinzipiell erfüllt, aber Daten für MAK- oder BAT-Wert-Ableitung nicht ausreichend

Hinweis: Formaldehydabspalter

Es liegt eine Begründung von 1991 (Henschler 1991) vor. Es erfolgt eine Neubewertung der Daten zur Formaldehydabspaltung nach aktuellem Vorgehen der Kommission (DFG 2024). Unter diesem Aspekt werden in diesem Nachtrag alle bewertungsrelevanten Endpunkte reevaluiert.

1,3-Bis(hydroxymethyl)harnstoff wird in der Regel als Pulver vertrieben. Der Gesamtformaldehydanteil beträgt ca. 46 %. Der Gehalt an freiem Formaldehyd wird mit weniger als 1 % angegeben (siehe Henschler 1991). Aus einem Molekül 1,3-Bis(hydroxymethyl)harnstoff entstehen zwei Moleküle Formaldehyd und ein Molekül Harnstoff. Zur Hydrolysegeschwindigkeit liegen keine Daten vor.

## Allgemeiner Wirkungscharakter

Der vermutlich kritische Effekt von 1,3-Bis(hydroxymethyl)harnstoff ist die kanzerogene und reizende Wirkung von freigesetztem Formaldehyd am Atemtrakt. An Haut und Auge des Kaninchens ist erst nach längerer Einwirkung eine Irritation zu erkennen. 1,3-Bis(hydroxymethyl)harnstoff wurde in zwei Mutagenitätstests an Salmonella typhimurium als mutagen bewertet. In einem HPRT-Test an L5178Y-Maus-Lymphomzellen wurde nur in Abwesenheit metabolischer Aktivierung ein schwach positives Ergebnis erhalten. Auch ein Mikronukleustest an V79-Zellen ist nur ohne Zusatz eines metabolischen Aktivierungssystems positiv. Ein Mikronukleustest am Knochenmark von NMRI-Mäusen sowie ein Comet-Assay in Leber, Magen und Duodenum von Sprague-Dawley-Ratten verliefen negativ. Es liegen keine Daten zur hautsensibilisierenden Wirkung von 1,3-Bis(hydroxymethyl)harnstoff beim Menschen vor. Ein Local Lymph Node Assay an der Maus verlief negativ.

Untersuchungen zur Toxizität nach wiederholter Gabe, zur Kanzerogenität und zur Entwicklungstoxizität liegen nicht vor.

## Wirkungsmechanismus

1,3-Bis(hydroxymethyl)harnstoff ist ein Formaldehydabspalter. Dies erklärt seine genotoxischen Effekte.

## Toxikokinetik und Metabolismus

Über die Stabilität und Zerfallskinetik liegen keine Angaben vor. Zur dermalen Aufnahme von 1,3-Bis(hydroxymethyl)harnstoff liegen keine experimentellen Untersuchungen vor. Abschätzungen auf der Basis von Modellrechnungen nach Fiserova-Bergerova et al. (1990) und IH SkinPerm v2.04 (Tibaldi et al. 2014) lassen für eine gesättigte wässrige Lösung von 130 g/l eine Resorption von 97 mg (0,807 mmol) bzw. 17 mg (0,14 mmol) unter Standardbedingungen (2000 cm^2^, eine Stunde) erwarten. 

## Tierexperimentelle Befunde und In-vitro-Untersuchungen

### Allergene Wirkung

#### Hautsensibilisierende Wirkung

In einem Local Lymph Node Assay nach OECD-Prüfrichtlinie 429 wurden mit Konzentrationen von 5, 10 und 25 % (höchstmögliche lösliche Konzentration) in DMSO Stimulationsindices von 1,08; 1,03 und 1,21 beobachtet. Das Testergebnis ist damit negativ (ECHA 2020).

#### Atemwegssensibilisierende Wirkung

Es liegen keine Untersuchungen zur atemwegssensibilisierenden Wirkung vor.

### Genotoxizität

Hierzu lagen in der bisherigen Begründung (Henschler 1991) keine Untersuchungen vor.

#### In vitro

In einem Mutagenitätstest nach OECD-Prüfrichtlinie 417 an Salmonella typhimurium TA98, TA100, TA102, TA1535 und TA1537 sowie Escherichia coli WP2 uvrA wurde 1,3-Bis(hydroxymethyl)harnstoff bis zu zytotoxischen Konzentrationen von etwa 2810 µg/Platte in An- und Abwesenheit eines Metabolisierungssystems untersucht. In dem Stamm TA102 war in An- und Anwesenheit des metabolischen Aktivierungssystems ein leichter, konzentrationsabhängiger Anstieg der Revertantenzahlen zu beobachten. 1,3-Bis(hydroxymethyl)harnstoff wurde als mutagen bewertet (ECHA 2020).

In einem zweiten Mutagenitätstest wurde 1,3-Bis(hydroxymethyl)harnstoff an Salmonella typhimurium TA98, TA100 und TA102 mit der Präinkubationsmethode und Konzentrationen von 0,21 bis 8,33 µmol/Platte in An- und Abwesenheit eines Metabolisierungssystems getestet. Zytotoxizität wurde bei den Stämmen TA98 und TA100 ohne metabolische Aktivierung bei 8,33 µmol/Platte beobachtet. Ein konzentrationsabhängiger Anstieg an Revertanten trat bei allen Stämmen in An- und Abwesenheit eines Metabolisierungssystems auf und die Substanz wurde als mutagen bewertet (ECHA 2020).

Ein HPRT-Test an L5178Y-Mauslymphomzellen wurde mit 1,3-Bis(hydroxymethyl)harnstoff nach OECD-Prüfrichtlinie 476 durchgeführt. Im ersten Experiment wurden Konzentrationen bis 900 µg/ml ohne Zusatz eines metabolischen Aktivierungssystems und bis 800 µg/ml mit metabolischer Aktivierung, sowie im zweiten Experiment bis 500 µg/ml ohne Zusatz eines metabolischen Aktivierungssystems geprüft. Das zweite Experiment wurde durchgeführt, um das schwach positive Ergebnis aus dem ersten Versuch zu überprüfen. Für alle Experimente wurde eine Inkubationszeit von drei Stunden angesetzt und die höchste Konzentration wirkte jeweils zytotoxisch. In Abwesenheit des metabolischen Aktivierungssystems wurde im ersten Experiment ein statistisch signifikanter Anstieg der Mutantenhäufigkeit bei der höchsten untersuchten Konzentration (900 µg/ml) beobachtet und es gab einen statistisch signifikanten linearen Trend (p ≤ 0,01). Einzelne Werte bei niedrigeren Konzentrationen überstiegen ebenfalls den historischen Kontrollbereich, was auf ein positives Ergebnis hindeutete. Im zweiten Experiment kam es zu einer Verschiebung der Toxizität, und nur drei Konzentrationen von 200 bis 500 µg/ml ergaben mehr als 10 % relatives Überleben. Es wurde ein statistisch signifikanter Anstieg der Mutantenhäufigkeit bei der höchsten Konzentration von 500 µg/ml beobachtet und es lag ein statistisch signifikanter linearer Trend vor (p ≤ 0,01). In Anwesenheit eines metabolischen Aktivierungssystems wurde bis zu zytotoxischen Konzentrationen kein statistisch signifikanter Anstieg der Mutantenhäufigkeit beobachtet und es gab auch keinen statistisch signifikanten linearen Trend. Das Ergebnis wurde als positiv gewertet (ECHA 2020).

Ein Mikronukleustest wurde mit 1,3-Bis(hydroxymethyl)harnstoff in Konzentrationen von 3 bis 333 µmol/l in An- und Abwesenheit eines metabolischen Aktivierungssystems an V79-Zellen durchgeführt. Jede Konzentration wurde zweimal unabhängig getestet und jeweils 500 Zellen ausgewertet. Die Zytotoxizität wurde mittels der relativen Dichte der Zellen bestimmt. 1,3-Bis(hydroxymethyl)harnstoff induzierte ohne Zusatz eines metabolischen Aktivierungssystems bei 100 und 333 µmol/l Mikronuklei und wirkte bei 333 µmol/l zytotoxisch. In Anwesenheit eines metabolischen Aktivierungssystems wurden weder zytotoxische noch klastogene oder aneugene Effekte beobachtet (ECHA 2020).

#### In vivo

Ein Comet-Assay wurde nach OECD-Prüfrichtlinie an männlichen Sprague-Dawley-Ratten nach zweimaliger, im Abstand von 21 Stunden oral verabreichter Gabe von 500, 1000 oder 2000 mg 1,3-Bis(hydroxymethyl)harnstoff/kg KG per Schlundsonde durchgeführt. Es wurden jeweils sechs Tiere pro Dosisgruppe und drei Tiere in der Positivkontrollgruppe eingesetzt und 24 Stunden nach der ersten Substanzgabe Gewebeproben aus Leber, Magen und Duodenum entnommen und auf DNA–Schäden untersucht. Als Vehikel wurde deionisiertes Wasser eingesetzt. 1,3-Bis(hydroxymethyl)harnstoff führte in keinem der Ansätze verglichen mit den Vehikelkontrollen zu einem statistisch signifikanten Anstieg des prozentualen Anteils fragmentierter DNA. Die Positivkontrolle zeigte ein funktionierendes Testsystem an. Es wurde daher geschlossen, dass 1,3-Bis(hydroxymethyl)harnstoff nicht zu einem signifikanten Anstieg der DNA-Schäden in Leber, Magen und Duodenum führt (ECHA 2020).

Mit 1,3-Bis(hydroxymethyl)harnstoff wurde ein Mikronukleustest nach OECD-Prüfrichtlinie 474 an den polychromatischen Erythrozyten des Knochenmarks von männlichen NMRI-Mäusen durchgeführt. Jeweils sieben Tiere pro Gruppe erhielten einmalig 500, 1000 oder 2000 mg 1,3-Bis(hydroxymethyl)harnstoff/kg KG per Schlundsonde verabreicht und wurden nach 24 oder 48 Stunden untersucht. Einige Tiere der Hochdosisgruppe hatten gesträubtes Fell. In den Vehikel- und Positivkontrollgruppen wurden jeweils nur fünf Tiere eingesetzt. Pro Tier wurden 2000 polychromatische Erythrozyten (PCE) auf Mikronuklei ausgewertet. Um eine zytotoxische Wirkung aufgrund der Behandlung zu untersuchen, wurde das Verhältnis von PCE zu normochromatischen Erythrozyten (NCE) in derselben Probe bestimmt. Nach der Behandlung mit 1,3-Bis(hydroxymethyl)harnstoff war die mittlere Anzahl der PCE pro 2000 Erythrozyten in der Hochdosisgruppe nach 24 und 48 Stunden etwas niedriger als der Mittelwert der Vehikelkontrolle. Allerdings lagen nur wenige Einzelwerte in der Hochdosisgruppe minimal unter dem niedrigsten Einzelwert der Vehikelkontrolle, was darauf hinweist, dass 1,3-Bis(hydroxymethyl)harnstoff, wenn überhaupt, nur eine geringe zytotoxische Wirkung im Knochenmark ausübt. Im Vergleich zu den entsprechenden Vehikelkontrollen gab es in keiner der Gruppen, die 1,3-Bis(hydroxymethyl)harnstoff erhielten, eine biologisch relevante oder statistisch signifikante Erhöhung der Mikronukleushäufigkeit. Die Positivkontrolle zeigte ein funktionierendes Testsystem an. 1,3-Bis(hydroxymethyl)harnstoff wurde als nicht genotoxisch in diesem Testsystem bewertet (ECHA 2020).

#### Fazit 

1,3-Bis(hydroxymethyl)harnstoff wurde in zwei Mutagenitätstests an Salmonella typhimurium, davon einer nach OECD-Prüfrichtlinie, als mutagen bewertet. In einem HPRT-Test nach OECD-Prüfrichtlinie an L5178Y-Mauslymphomzellen wurde nur in Abwesenheit metabolischer Aktivierung ein schwach positives Ergebnis erhalten. Auch ein Mikronukleustest an V79-Zellen war nur ohne Zusatz eines metabolischen Aktivierungssystems positiv. In vivo verliefen ein Mikronukleustest nach OECD-Prüfrichtlinie 474 an den polychromatischen Erythrozyten des Knochenmarks männlicher NMRI-Mäuse sowie ein Comet-Assay in Leber, Magen und Duodenum männlicher Sprague-Dawley-Ratten, jeweils nach oraler Gabe von bis zu 2000 mg 1,3-Bis(hydroxymethyl)harnstoff/kg KG, negativ (ECHA 2020).

Auch Formaldehyd ist nach oraler Gabe negativ im Maus-Mikronukleustest, aber positiv mit hohen intraperitonealen Dosen (Greim 2000), so dass trotz der hier vorliegenden negativen oralen In-vivo-Daten eine keimzellmutagene Wirkung von 1,3-Bis(hydroxymethyl)harnstoff nach Inhalation nicht völlig ausgeschlossen werden kann. 

### Kanzerogenität

Hierzu liegen keine Daten vor. 

## Bewertung

Kritische Effekte sind die kanzerogene und lokal reizende Wirkung des Hydrolyseprodukts Formaldehyd.

**Krebserzeugende Wirkung. **Es liegen keine Untersuchungen der krebserzeugenden Wirkung von 1,3-Bis(hydroxymethyl)harnstoff vor. 1,3-Bis(hydroxymethyl)harnstoff selbst weist nur in vitro ein genotoxisches Potenzial auf, eine mögliche genotoxische Wirkung am wahrscheinlichen Zielgewebe des oberen Atemtrakts bzw. der Nase (wie bei Formaldehyd) ist jedoch nicht untersucht.

1,3-Bis(hydroxymethyl)harnstoff hydrolysiert zu Formaldehyd und Harnstoff. Aufgrund des Fehlens von Daten zur Hydrolysegeschwindigkeit wird als Worst-Case-Abschätzung davon ausgegangen, dass 1,3-Bis(hydroxymethyl)harnstoff beim Auftreffen im Atemtrakt sofort vollständig zu Formaldehyd und Harnstoff hydrolysiert und der Abbau des aus 1,3-Bis(hydroxymethyl)harnstoff entstehenden Formaldehyds langsamer erfolgt als dessen Bildung.

Harnstoff ist ein endogenes Stoffwechselprodukt. Eine mögliche kanzerogene Wirkung von exogenem Harnstoff kann anhand der vorliegenden Datenlage nicht abschließend bewertet werden (Dickerson et al. 2018).

Die lokale Kanzerogenität des Hydrolyseprodukts Formaldehyd hingegen ist ausführlich dokumentiert (Greim 2000; Hartwig 2010). Formaldehyd ruft Nasentumoren hervor, jedoch erst bei Konzentrationen, die die Entgiftungskapazitäten des Nasengewebes überschreiten. Daher ist Formaldehyd in Kanzerogenitäts-Kategorie 4 eingestuft.

Aufgrund der lokalen kanzerogenen Wirkung von Formaldehyd könnte 1,3-Bis(hydroxymethyl)harnstoff in Analogie zu Formaldehyd in Kanzerogenitäts-Kategorie 4 eingestuft werden. Da jedoch kein MAK-Wert für 1,3-Bis(hydroxymethyl)harnstoff abgeleitet werden kann, wird der Stoff der Kanzerogenitäts-Kategorie 2 zugeordnet und erhält die Fußnote „Voraussetzung für Kategorie 4 prinzipiell erfüllt, aber Daten für MAK- oder BAT-Wert-Ableitung nicht ausreichend“.

**MAK-Wert. **Es liegen keine Inhalationsstudien mit 1,3-Bis(hydroxymethyl)harnstoff am Menschen oder am Tier vor, aus denen ein MAK-Wert abgeleitet werden kann.

1,3-Bis(hydroxymethyl)harnstoff ist ein Formaldehydabspalter. Formaldehyd zeigt deutliche Reizwirkungen in der Nase und kann bei chronischer Exposition Tumoren im Epithel der Nasenschleimhaut erzeugen. Die Entgiftungskapazitäten des Nasengewebes für Formaldehyd werden bei einem MAK-Wert von 0,3 ml/m^3^ (0,37 mg/m^3^) nicht überschritten (Greim 2000; Hartwig 2010).

Der Dampfdruck von 1,3-Bis(hydroxymethyl)harnstoff (< 1 × 10^–6^ hPa) zeigt, dass der Stoff bei Konzentrationen, bei denen Formaldehyd schon dampfförmig ist (Dampfdruck 5185 hPa (OECD 2002)), noch als Aerosol vorliegt. Für Aerosole ist durch die Impaktierung im Atemtrakt mit einer stärkeren Wirkung als die durch dampfförmiges Formaldehyd verursachte zu rechnen, wie mit einem anderen Formaldehydabspalter, zu dem eine Inhalationsstudie vorliegt, gezeigt wurde (siehe Begründung N,N′,N′′-Tris(β-hydroxyethyl)hexahydro-1,3,5-triazin; Hartwig und MAK Commission 2023 b).

Für 1,3-Bis(hydroxymethyl)harnstoff kann somit kein MAK-Wert festgesetzt werden. Eine Spitzenbegrenzung entfällt.

Bei Anwendung in verdünnten wässrigen Lösungen sollte mit einer vollständigen Hydrolyse gerechnet und daher der MAK-Wert für Formaldehyd (Greim 2000; Hartwig 2010) eingehalten werden.

**Fruchtschädigende Wirkung. **Es liegen keine Daten zur fruchtschädigenden Wirkung vor. Da kein MAK-Wert aufgestellt werden kann, entfällt die Zuordnung zu einer Schwangerschaftsgruppe.

**Keimzellmutagene Wirkung. **1,3-Bis(hydroxymethyl)harnstoff ist in vitro genotoxisch im Salmonella-Mutagenitätstest, im HPRT-Test an L5178Y-Mauslymphomzellen und im Mikronukleustest an V79-Zellen, nicht jedoch in vivo im Mikronukleustest an Knochenmarkszellen der Maus und im Comet-Assay an Leber, Magen und Duodenum der Ratte. Untersuchungen an Keimzellen liegen nicht vor.

Wird davon ausgegangen, dass nach Inhalation durch Hydrolyse sofort das gesamte Formaldehyd im oberen Atemtrakt freigesetzt wird, ist dieses wahrscheinlich nicht systemisch verfügbar. Daten dazu fehlen jedoch.

Theoretisch könnte 1,3-Bis(hydroxymethyl)harnstoff in Analogie zu Formaldehyd in die Kategorie 5 für Keimzellmutagene (Greim 2000) eingestuft werden, jedoch kann für 1,3-Bis(hydroxymethyl)harnstoff kein MAK-Wert aufgestellt werden. 

Da Daten zur systemischen Bioverfügbarkeit von 1,3-Bis(hydroxymethyl)harnstoff und dem durch Hydrolyse freigesetzten Formaldehyd fehlen, liegt kein experimenteller Beleg vor, dass der freigesetzte Formaldehyd in aktiver Form die Keimzellen erreicht. Daher wird 1,3-Bis(hydroxymethyl)harnstoff in Kategorie 3 B für Keimzellmutagene eingestuft.

**Hautresorption. **Zur dermalen Aufnahme von 1,3-Bis(hydroxymethyl)harnstoff liegen keine experimentellen Untersuchungen vor. Abschätzungen auf der Basis von Modellrechnungen lassen eine Resorption von maximal 97 mg (0,807 mmol) innerhalb einer Stunde erwarten. Eine Markierung mit „H“ kann hieraus aufgrund fehlender Daten zum systemischen NOAEL nicht direkt abgeleitet werden. 

Unter der Annahme einer raschen vollständigen Hydrolyse mit Bildung von zwei Molekülen Formaldehyd pro Molekül 1,3-Bis(hydroxymethyl)harnstoff lässt sich die Zunahme des Formaldehydspiegels im Blut nach dermaler Applikation abschätzen: aus den oben berechneten Daten ergibt sich über einen Zeitraum von einer Stunde eine maximale Freisetzung von 49 mg Formaldehyd (2 × 0,807 mmol). Pro Minute werden demnach etwa 810 µg bzw. 1012 µg/1,25 Minuten (mittlere Halbwertszeit des Formaldehyds im Blut; Kaden et al. 2010) freigesetzt. Die transdermale Aufnahme von 1,3-Bis(hydroxymethyl)harnstoff in den letzten sechs Halbwertszeit-Intervallen führt demnach zu einer im Blut zirkulierenden Formaldehydmenge von (1012 + 506 + 253 + 127 + 64 + 32) µg = 1994 µg bzw. etwa 2 mg. Der physiologisch bedingte Formaldehydspiegel im Blut des Menschen beträgt etwa 2–3 mg/l (10–15 mg in 5 Liter Blut, frei und reversibel gebunden) (Heck et al. 1985). Dieser zusätzliche Eintrag von Formaldehyd durch transdermal aufgenommenen 1,3-Bis(hydroxymethyl)harnstoff erhöht demnach die Gleichgewichtskonzentration von Formaldehyd im Blut unbedeutend, so dass 1,3-Bis(hydroxymethyl)harnstoff weiterhin nicht mit „H“ markiert wird.

**Sensibilisierende Wirkung. **Die Auslösung einer Formaldehyd-Sensibilisierung durch 1,3-Bis(hydroxymethyl)harnstoff kann aufgrund der Eigenschaft der Substanz, Formaldehyd freizusetzen, nicht ausgeschlossen werden. Dennoch liegen weiterhin keine Daten zur hautsensibilisierenden Wirkung beim Menschen vor. Ein Local Lymph Node Assay verlief bis zu einer Konzentration von 25 % negativ. Es erfolgt daher weiterhin keine Markierung mit „Sh“. Daten zur sensibilisierenden Wirkung an den Atemwegen fehlen, sodass weiterhin keine Markierung mit „Sa“ erfolgt.

## References

[ref_86CDE6IJ] Deutsche Forschungsgemeinschaft DFG (2024). MAK- und BAT-Werte-Liste 2024. Maximale Arbeitsplatzkonzentrationen und Beurteilungswerte in biologischem Material. Ständige Senatskommission zur Prüfung gesundheitsschädlicher Arbeitsstoffe, Mitteilung 60. Erratum..

[ref_WLZWALCH] Dickerson Aisha S., Lee Janice S., Keshava Channa, Hotchkiss Andrew, Persad Amanda S. (2018). Assessment of health effects of exogenous urea: summary and key findings. Curr Environ Health Rep.

[ref_7AA4MMDC] (2020). 1,3-Bis(hydroxymethyl)urea (CAS Number 140-95-4). Registration dossier. Joint submission, first publication 02 May 2018, last modification 06 Jul 2020.

[ref_K3MNQIRK] European Commission (2013). Existing active substances for which a decision of non-inclusion into Annex I or Ia of Directive 98/8/EC has been adopted.

[ref_WIZRBQ2Y] Fiserova-Bergerova V., Pierce J. T., Droz P. O. (1990). Dermal absorption potential of industrial chemicals: criteria for skin notation. Am. J. Ind. Med..

[ref_X4PKN6YQ] Greim H (2000).

[ref_C9HFUEAQ] Hartwig A (2010).

[ref_WUZKU3QW] Hartwig A., MAK Commission (2023). Komponenten von Kühlschmierstoffen, Hydraulikflüssigkeiten und anderen Schmierstoffen. MAK-Begründung, Nachtrag.. MAK Collect Occup Health Saf.

[ref_QRKIUM76] Hartwig A., MAK Commission (2023). N,N′,N′′-Tris(β-hydroxyethyl)hexahydro-1,3,5-triazin. MAK-Begründung, Nachtrag. MAK Collect Occup Health Saf.

[ref_57MCISV2] Heck H., Casanova-Schmitz M., Dodd P. B., Schachter E. N., Witek T. J., Tosun T. (1985). Formaldehyde (CH_2_O) concentrations in the blood of humans and Fischer-344 rats exposed to CH_2_O under controlled conditions. Am Ind Hyg Assoc J.

[ref_B6REZ2M8] Henschler D (1991).

[ref_WL9ES85T] Kaden D.A., Mandin C., Nielsen G.D., Wolkoff P., World Health Organization WHO (2010). WHO guidelines for indoor air quality: selected pollutants.

[ref_JUGW727Q] Organisation for Economic Co-operation and Development OECD (2002). Formaldehyde (CAS No 50-00-0). OECD SIDS Initial Assessment Report.

[ref_7C2FXEEC] Tibaldi Rosalie, ten Berge Wil, Drolet Daniel (2014). Dermal absorption of chemicals: estimation by IH SkinPerm. J Occup Environ Hyg.

